# Incidence and Risk Factors of Arytenoid Dislocation Following Endotracheal Intubation: A Systematic Review and Meta-Analysis

**DOI:** 10.7759/cureus.67917

**Published:** 2024-08-27

**Authors:** Nasser Saad Alalyani, Alhanouf Abdulaziz Alhedaithy, Hind Khaled Alshammari, Rafeef I AlHajress, Rakan H Alelyani, Malak Fawaz Alshammari, Abdullah Hassan Alhalafi, Amani Alharbi, Nada Aldabal

**Affiliations:** 1 Department of Otolaryngology, Head and Neck Surgery, King Fahd Military Medical Complex, Dhahran, SAU; 2 Department of Otolaryngology, Head and Neck Surgery, Johns Hopkins Aramco Healthcare, Dhahran, SAU; 3 Department of Otolaryngology, Head and Neck Surgery, King Saud University Medical City, Riyadh, SAU; 4 College of Medicine, King Abdulaziz University Hospital, Jeddah, SAU; 5 College of Medicine, Imam Mohammad Ibn Saud Islamic University, Riyadh, SAU; 6 Department of Family and Community Medicine, College of Medicine, University of Bisha, Bisha, SAU

**Keywords:** laryngeal trauma, systematic review and meta analysis, risk factors, endotracheal intubation, arytenoid dislocation

## Abstract

Endotracheal intubation carries risks, including arytenoid dislocation (AD), a rare but severe complication. Due to small sample sizes, the incidence of AD varies considerably among studies. Proposed risk factors for AD include difficult intubation, prolonged intubation, certain surgeries, patient positioning, female sex, and BMI. This review aims to investigate the incidence of AD and explore the various predisposing risk factors.

We retrieved relevant studies up to April 2024 from PubMed, Scopus, Web of Science, and the Cochrane Library. Using OpenMeta v5.26.14 software (Institute for Clinical Research and Health Policy Studies at Tufts Medical Center, Boston, USA), we pooled AD incidence rates from individual studies. Other outcomes, reported in fewer studies and thus not suitable for meta-analysis, were synthesized manually.

Study selection yielded 16 eligible articles. A random-effects model analysis of nine studies found a significant AD incidence rate of 0.093% (confidence interval (CI): 0.045% to 0.14%), but the results were highly heterogeneous (I^2^ = 91%). Older age was associated with prolonged hoarseness, while younger age and female sex increased the risk of AD. Additionally, AD risk factors included taller stature, higher BMI, specific surgeries, esophageal instrumentation, prolonged procedure durations, head-neck movement, and inexperienced intubators. However, intubation with a stylet reduced the AD risk.

AD post-endotracheal intubation is rare (incidence: 0.09%), with potential underdiagnosis in larger datasets. Many risk factors may contribute to the condition, but the small number of studies per risk factor limits the ability to draw robust conclusions. Subjective diagnoses and retrospective studies further restrict comprehensive understanding. Further research is needed to explore AD risk factors effectively.

## Introduction and background

Endotracheal intubation is a common procedure in various medical settings, such as intensive care units (ICUs), emergency departments, and during surgeries. It involves inserting an endotracheal tube into the trachea to secure the patient’s airway and facilitate mechanical ventilation [1-3]. Despite its critical importance, complications during endotracheal intubation remain a significant concern. In ICUs, complication rates have been reported to range from 25% to 39%, while emergency intubations in a university hospital showed incidence rates ranging from 4.1% to 28% [1,4]. These complications can range from common issues like hypoxia and hypotension to more severe ones such as dysrhythmias, aspiration, and incorrect tube placement [2]. Among the rarer but more severe complications are arytenoid dislocation (AD), tooth aspiration, and uvular necrosis [5-7].

AD is a rare yet major complication that can occur following airway procedures, including endotracheal intubation and laryngeal trauma [8,9]. First reported in 1973, AD involves the displacement of the arytenoid cartilage, which plays a crucial role in laryngeal function and vocal cord movement [10,11]. The incidence of post-intubation AD ranges from 0.01% to 0.904% per 100,000 cases [8]. Risk factors for AD have been extensively studied, identifying several critical contributors: difficult intubation, prolonged intubation duration, major cardiovascular surgeries, patient positioning, female sex, BMI, the use of airway tools, and various airway procedures [8,12-18].

The manifestations of AD can vary in severity, affecting vocal function, swallowing, and overall laryngeal health [13]. Common symptoms include hoarseness, throat pain or discomfort, and dysphagia [18,19]. Patients may also experience a choking cough due to altered laryngeal anatomy and function. In severe cases, dyspnea may occur due to airway compromise [18]. AD should be suspected following endotracheal intubation if patients exhibit persistent hoarseness, aspiration, or vocal disability post-procedure [9,18,20]. Clinical signs indicative of AD during direct laryngoscopy include joint space asymmetry, obliteration or widening, and the absence of the “jostle sign,” which refers to the passive medial movement of the paralyzed vocal cord during adduction [21,22]. Healthcare providers should consider AD as a potential diagnosis in cases of postoperative hoarseness, particularly after ruling out conditions like laryngeal nerve injuries [23,24].

Treatment options for AD include both conservative and surgical approaches, depending on the severity and response to initial management. Surgical procedures such as closed reduction, thyroplasty, arytenoid adduction/rotation, arytenoidopexy, and injection laryngoplasty are employed to stabilize the arytenoid cartilage and restore laryngeal function [14,25]. Early intervention is critical for optimal outcomes, as prompt diagnosis and treatment can prevent complications, improve prognosis, avoid cricoarytenoid joint ankylosis, and preserve joint mobility, all of which are essential for normal laryngeal function and voice quality [7,14,25].

Given its infrequent occurrence, it is crucial to gather evidence from all available sources to thoroughly understand the incidence and risk factors associated with AD. Our review aims to investigate the occurrence of AD following endotracheal intubation and to explore the various factors that may contribute to its development. By doing so, we strive to enhance patient care and safety through a comprehensive analysis of this rare condition.

## Review

Materials and methods

In this review, we followed the guidelines outlined by the Preferred Reporting Items for Systematic Reviews and Meta-Analyses (PRISMA) and the Cochrane Handbook for Systematic Reviews of Interventions [26,27].

Eligibility Criteria

The inclusion criteria consisted of original research articles written in English, involving human samples, and containing all required data, including details on AD patients and risk factors associated with endotracheal intubation. Exclusion criteria included abstracts, letters to editors, comments, reviews, and articles not written in English.

Information Sources and Search Strategy

The record retrieval process involved several stages. Initially, we searched PubMed and Scopus using generic terms to identify relevant articles. We then developed a search strategy incorporating all pertinent terms identified in the initial search, along with their corresponding MeSH terms. In the second stage, we expanded our search to include PubMed, Scopus, Cochrane Library, and Web of Science using specific search strings related to AD and endotracheal intubation. The final search strategy was as follows: (Arytenoid dislocation OR AD OR Arytenoid cartilage dislocation) AND (Endotracheal intubation OR Fiberoptic intubation OR Intubation). The search was conducted in April 2024. Finally, we manually examined the references and citations of the retrieved records to identify additional relevant studies.

Selection Process

Two reviewers independently screened the titles and abstracts of all available records based on the predefined eligibility criteria, with any conflicts reviewed by a third reviewer. Following this, the same reviewers assessed the full texts of the articles included in the previous step, resolving disputes through discussion.

Data Collection and Outcome Variables

Two independent reviewers extracted relevant data into an Excel sheet (Microsoft® Corp., Redmond, USA) with predefined variables. The data extraction process involved gathering pertinent information from the included studies, such as general details (e.g., the first author’s name, study design, country of origin) and demographic characteristics of patients with AD, including the number of patients, their age, BMI, and gender distribution. Additionally, data regarding the operative procedure were collected, including types of surgery, tube size distribution, duration of surgery, intubation time, tracheal intubation tools used, patient positioning, number of intubation attempts, American Society of Anesthesiologists (ASA) physical status, stylet use, types of dislocation, and the side of AD. These variables were analyzed to understand the risk factors associated with AD and its prevalence. Finally, conclusions drawn from the studies were summarized to provide insights into the findings regarding AD.

Risk of Bias

Two independent reviewers assessed the quality of eligible articles, resolving conflicts through discussion. We evaluated the methodological quality of cohort and case-control studies using the National Institutes of Health (NIH) Quality Assessment Tool for Observational Cohort Studies [28].

Statistical Analysis

We conducted the analysis using OpenMeta v5.26.14 software (Institute for Clinical Research and Health Policy Studies at Tufts Medical Center, Boston, USA). This meta-analysis aimed to determine the overall prevalence of AD and the corresponding 95% confidence interval (CI). Statistical significance was defined as a P-value less than 0.05. Heterogeneity among the included studies was assessed using the I² statistic, with 25%, 50%, and 75% indicating low, moderate, and high heterogeneity, respectively. Heterogeneity was further evaluated using additional Tau² and Q-test methods. Significant heterogeneity was defined as an I² exceeding 50% along with a P-value of less than 0.1. We employed the random effects model to address variations between populations and operative procedures. The results were visualized in a forest plot that included information on individual studies and the heterogeneity of the effect measure.

Results

Study Selection

**Figure 1:**
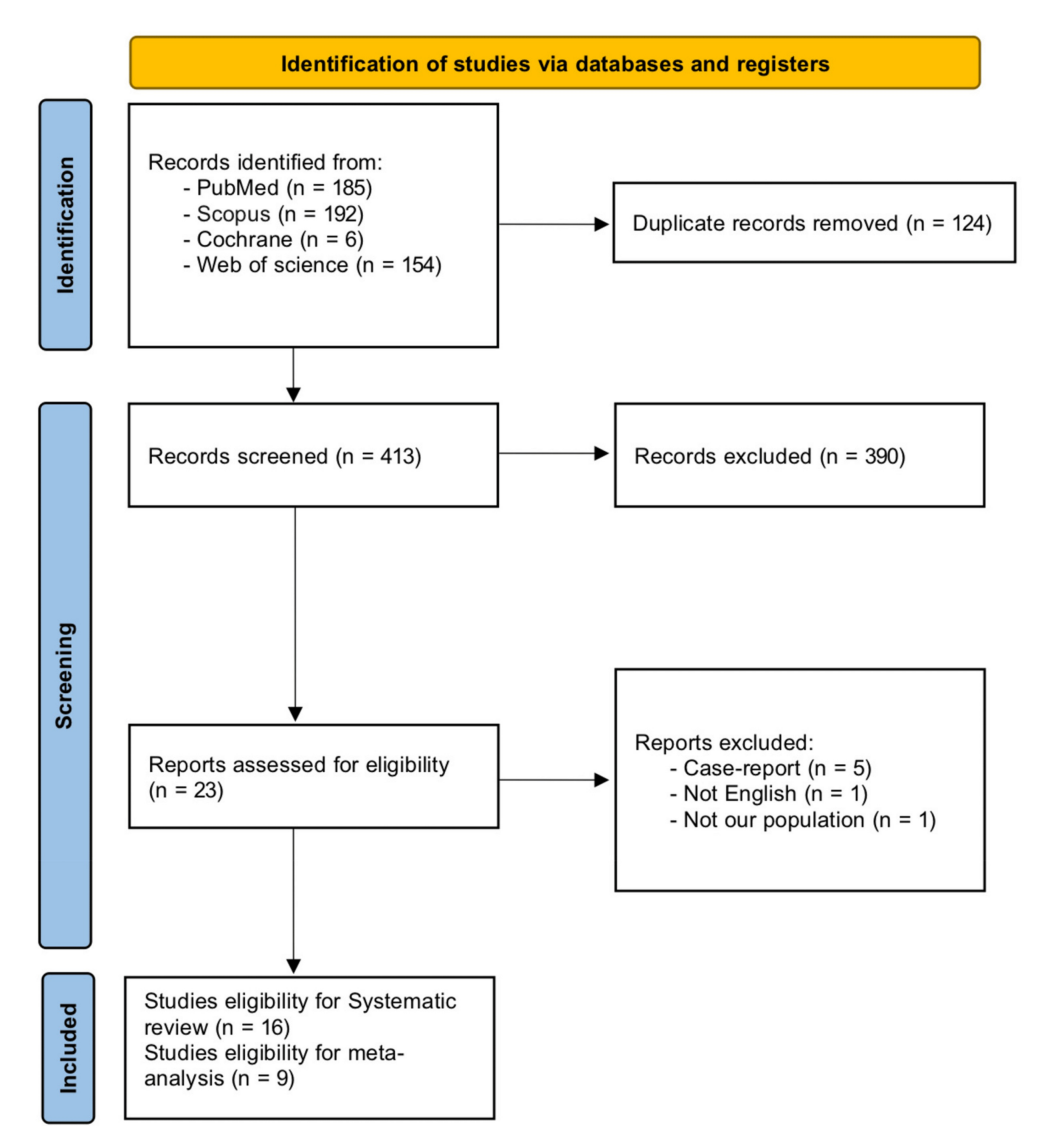
PRISMA chart of selected studied PRISMA: Preferred Reporting Items for Systematic Reviews and Meta-Analyses

After employing the search strategy in the four databases, we obtained 537 records. Of these, 124 duplicates were excluded, resulting in 413 unique records. Screening the titles and abstracts of these records led to the exclusion of 390 entries. Next, we retrieved the full texts of the remaining 23 records and assessed them thoroughly against our eligibility criteria. During this process, five additional articles were excluded. Finally, the review included 16 articles, of which nine had sufficient data for analysis. Figure 1 displays the flow diagram for study selection.

Characteristics of the Included Studies

Eligible articles comprised eight retrospective cohort studies, seven case-control studies, and one prospective cohort study [8,9,13,15,17,20,25,29-37]. Overall, 366 cases of AD were reported across these studies. The studies were primarily from China (nine), followed by Japan (three), South Korea (two), the USA (one), and Taiwan (one). Fourteen articles reported the ages of patients with AD, averaging 51.2 years, and the BMI averaged 24.28 kg/m² according to eight articles reporting this information. As for gender distribution, 174 (47.5%) patients were male, and 192 (52.5%) were female. The intubation instruments used included conventional laryngoscopy (three studies), video laryngoscopy (four studies), both conventional and video laryngoscopy (four studies), both conventional and fiberoptic laryngoscopy (three studies), and fiberoptic laryngoscopy alone (two studies). Detailed baseline data are presented in Table 1.

**Table 1:** Summary and baseline characteristics of the included studies NR: not reported; NA: not applicable; Y: year; AD: arytenoid dislocation; N: number; SD: standard deviation; ASA: American Society of Anesthesiologists classification

Study ID	Study design	Country	No. of patients with or without AD	Age, mean ± SD	BMI, kg/m^2^	Male, N (%)	Type of the surgery, N (%)	Tube size in mm, N (%)	Duration of surgery, h	Intubation time, min	Tracheal intubation tool	Position	No. of intubation attempts	ASA physical status	Stylet use	Type of dislocation, N (%)	Arytenoid dislocation side, N (%)	Conclusion
Hung et al., 2019 [20]	Case-control study	Taiwan	AD, 14	39.9 ± 10.3	35.2 ± 9.1	6 (42.9%)	Bariatric/Metabolic surgery = 14 (100%)	7 mm = 5 (35.71%); 7.5 mm = 8 (57.14%); 8 mm = 1 (7.15%)	NR	235 ± 133	Fibreoptic laryngoscopy or Video-laryngoscopy	NR	One attempt= 14 (100%)	NR	NR	NR	Left = 13 (93%); Right = 1 (7%)	"Our report demonstrates that the clinical characteristics of patients with AD who received tracheal intubation and OG insertion for bariatric/metabolic surgery were different from those with postoperative AD receiving only tracheal intubation, highlighting the importance of implementing individualized strategies for AD prevention in this patient population."
			Non-AD, 1721	NR	NR	NR	NR	NR		NR	NR		NR			NA	NA	
Jang et al., 2021 [13]	Retrospective cohort study	South Korea	AD, 33	53.6 ± 14.4	24.2 ± 3.4	7 (22.2%)	Emergency surgery = 33 (100%)	6 mm = 1 (3.0%); 6.5 mm = 1 (3.0%); 7 mm = 26 (78.8%); 8 mm = 5 (15.2%)	2.86 ± 1.94	NR	Conventional laryngoscope = 33 (100%)	Supine= 26 (78.8%); Lateral = 7 (22.2%)	One attempt= 32 (97%); Two attempts = 1 (3%)	ASA I = 14 (42.4%); ASA II = 17 (51.5%); ASA III = 2 (6.1%)	1 (3%)	NR	NR	"This study showed that the incidence of arytenoid dislocation was 0.13% and that head-neck positioning during surgery, less anesthetist experience, and females were significantly associated with arytenoid dislocation in patients who underwent surgeries under general anesthesia with endotracheal intubation."
			Non-AD, 25505	58.2± 14.2	24 ± 3.6	11217 (43.5%)	Emergency surgery = 25505 (100%)	<6 mm = 14; 6 mm = 202 (0.8%); 6.5 mm = 260 (1%); 7 mm = 14526 (57%); 7.5 mm = 66 (0.3%); 8 mm = 10434 (40.9%); 8.5 mm = 2; 9 mm = 1	2.75 ± 1.96		Conventional laryngoscope = 25077 (98.3%), Video-laryngoscope = 327 (1.3%), Fibreoptic laryngoscopy = 18	Supine= 23448 (91.9%); Lateral = 1698 (6.7%); Prone = 359 (1.4%)	One attempt= 24794 (97.2%); Two attempts = 697 (2.7%); Three attempts = 13 (0.1%); Four attempts = 1	ASA I = 6587 (52.8%); ASA II = 16009 (62.8%); ASA III = 2845 (11.2%); ASA IV = 64 (0.3%)	939 (3.7%)	NA	NA	
Jiang et al., 2023 [29]	Retrospective cohort study	China	AD, 5	32.8 ± 7.2	20.24 ± 1.01	0	Facial bony contouring surgery = 5 (100%)	6.5 mm = 3 (60%); 7 mm = 2 (40%)	3.17 ± 0.66	1102 ± 115.2	Video-laryngoscope = 5 (100%)	Supine = 5 (100%)	One attempt= 5 (100%)	ASA I = 4 (80%); ASA II = 1 (20%)	5 (100%)	NR	NR	"Arytenoid dislocation may result from multiple factors instead of one high-risk factor. Head-neck movement, the skills and experience of anesthetists, the time of intubation, and the use of intubation tools may all predispose patients to arytenoid dislocation. To acquire timely diagnosis and treatment, patients should be fully informed of this complication before surgery and observed closely afterward. Any postoperative voice or laryngeal symptoms lasting more than seven days need a specialist evaluation."
			Non-AD, 436	29 ± 5	20.201 ± 2.03	37 (8.5%)	Facial bony contouring surgery = 436 (100%)	6 mm = 3 (0.7%); 6.5 mm = 292 (67%); 7 mm = 141 (32.3%)	3.01 ± 0.82	1012.4 ± 316	Video-laryngoscope = 242 (55.5%), Conventional-laryngoscope = 184 (42.4%)	Supine = 206 (47.2%)	One attempt= 436 (100%)	ASA I = 376 (86.2%); ASA II = 60 (13.8%)	436 (100%)	NA	NA	
Kong et al., 2022 [30]	Case-control study	China	AD, 49	56.7 ± 7.4	23.6 ± 3.97	24 (49%)	Abdominal/general surgery = 40 (82%); Cardiovascular surgery =2 (4%); Orthopedics surgery = 3 (6%); Gynecology = 1 (2%); Otolaryngology = 1 (2%); Urologic = 1 (2%); Oral = 1 (2%)	7 mm = 32 (65.4%); 7.5 mm = 15 (30.6%)	3.87 ± 2.85	NR	Conventional laryngoscope or Video-laryngoscope	NR	NR	NR	NR	Anterior = 48 (97.9%); Posterior = 1 (2.1%)	Left = 34 (69.4%); Right = 15 (30.6%)	"The use of an NG tube, abdominal surgery, and longer operative time were risk factors for AD. Among these, the NG tube application showed a strong association with AD. Preventive measures of informing the patients of the increased risk and providing high-level patient monitoring can reduce the incidence of AD."
			Non-AD, 245		24.1 ± 3.5	121 (49.4%)	Abdominal/general surgery = 79 (32.2%); Cardiovascular surgery =14 (5.7%); Orthopedics surgery = 56 (22.9%); Gynecology = 28 (11.4%); Otolaryngology = 29 (11.8%); Urologic = 18 (7.4%); Oral = 10 (4.1%); Neurosurgery = 11 (4.5%)	5 mm = 3 (1.2%); 6 mm = 4 (1.6%); 6.5 mm = 15 (6.1%); 7.5 mm = 105 (42.9%); 7.5 mm = 191 (37.1%)	2 ± 2.63							NA	NA	
Lee et al., 2015 [31]	Retrospective cohort study	South Korea	AD, 13	54.1 ± 15.9	NR	6 (46.2%)	Abdominal/general surgery = 4 (30.8%); Orthopedics surgery = 2 (15.4%); Head and Neck surgery = 7 (53.8%)	NR	3.95 ± 3.8	NR	Fiberoptic laryngoscopy	NR	NR	NR	NR	Anterior = 12 (92.3%); Posterior = 1 (7.7%)	Left = 8 (61.5%); Right = 5 (38.5%)	"This study indicates that the arytenoid dislocations after the endotracheal intubation may be needed for the aggressive surgical intervention, even if the diagnosis was delayed."
Liu et al., 2018 [32]	Retrospective cohort study	China	AD, 3	NR	NR	2 (66.67%)	Orthognathic surgery = 3 (100%)	6.5 mm = 2 (66.7%); 7 mm = 1 (33.3%)	NR	NR	Fiberoptic laryngoscopy	NR	NR	NR	NR	NR	NR	"Arytenoid dislocation must be considered in cases of prolonged hoarseness post-orthognathic surgery. Examination should be carried out as soon as possible, which can hasten the treatment of arytenoid dislocation and achieve a good outcome."
			Non-AD, 5029			NR	Orthognathic surgery = 5029 (100%)	NR			NR					NA	NA	
Lou et al., 2018 [15]	Retrospective cohort study	China	AD, 35	51.7	NR	21 (60%)	Abdominal/general surgery = 30 (85.6%); Cardiovascular surgery = 3 (8.6%); Orthopedics surgery = 1 (2.9%); Neurosurgery = 1 (2.9%)	NR	NR	NR	Conventional laryngoscope or Video-laryngoscope	NR	NR	NR	NR	Anterior = 32 (91.4%); Posterior = 3 (8.6%)	Left = 19 (54.3%); Right = 16 (45.7%)	"Based on the treatment outcomes from 34 patients with arytenoid dislocation, we have confirmed that closed reduction under local anesthesia is an effective and safe procedure. Performing closed reduction within a time window ranging from the 13th day to the 26th day after arytenoid dislocation caused by intubation can provide satisfactory treatment outcomes and short treatment duration. Thus, closed reduction performed in this appropriate time window is a practical and effective treatment option for arytenoid dislocation caused by intubation."
Lou and Lin, 2017 [34]	Case-control study	China	AD, 28	55 ± 12	20.33 ± 3.3	18 (64.3%)	Abdominal/general surgery = 21 (75%); Cardiovascular surgery = 4 (14.3%); Orthopedics surgery = 2 (7.1%); Neurosurgery = 1 (3.6%)	6-6.5 mm = 12 (42.85%); 7-7.5 mm = 16 (57.15%)	3.17 ± 1.72	NR	Video-laryngoscope	NR	NR	NR	28 (100%)	Anterior = 26 (92.9%); Posterior = 2 (7.1%)	Left = 16 (57%); Right = 12 (43%)	"BMI might be the independent risk factor for postoperative arytenoid dislocation."
			Non-AD, 56	53 ± 14	22.94 ± 3.02	40 (71.4%)	Abdominal/general surgery = 42 (75%); Cardiovascular surgery = 8 (14.3%); Orthopedics surgery = 4 (7.1%); Neurosurgery = 2 (3.6%)	6-6.5 mm = 23 (41%); 7-7.5 mm = 33 (59%)	3.7 ± 1.42		NR				56 (100%)	NA	NA	
Lou et al., 2023 [33]	Case-control study	China	AD, 28	51.8 ± 13.4	NR	18 (64.3%)	NR	NR	NR	NR	Conventional laryngoscope or Video-laryngoscope	NR	NR	NR	NR	Anterior = 27 (96.4%); Posterior = 1 (3.6%)	Left = 15 (53.6%); Right = 13 (46.4%)	"Closed reduction with the modified laryngeal forceps under local anesthesia is an effective and safe procedure. Compared with traditional laryngeal forceps, the modified laryngeal forceps can shorten the treatment duration."
Rubin et al., 2005 [25]	Retrospective cohort study	USA	AD, 63	42.5 ± 18.6	NR	24 (38.1%)	NR	NR	NR	NR	Conventional laryngoscope	NR	NR	NR	NR	Anterior = 17 (26%); Posterior = 32 (50.2%); Complex = 6 (9.5%); Both = 3 (4.8%); Not Reported = 6 (9.5%)	Left = 35 (55.5%); Right = 25 (39.7%); Bilateral = 3 (4.8%)	"The diagnosis of arytenoid cartilage dislocation should be considered in all cases of vocal fold hypomobility and immobility. Videostroboscopy is the most critical part of the examination, although laryngeal CT and EMG are useful to otolaryngologists. An attempt at endoscopic reduction should be considered even when diagnosis has been delayed. Equalizing the heights of the vocal processes might improve the voice even without the return of joint mobility."
Saigusa et al., 2013 [35]	Retrospective cohort study	Japan	AD, 20	66.9 ± 10.2	NR	13 (65%)	Abdominal/general surgery = 2 (10%); Cardiothoracic surgery = 15 (75%); Neurosurgery = 2 (10%); Others = 1 (5%)	NR	NR	NR	Video-laryngoscope	NR	NR	NR	NR	Anterior = 14 (70%); Posterior = 6 (30%)	Left = 15 (75%); Right = 5 (25%)	"It could be considered that additional medical instrumentation of the esophagus, including transesophageal echocardiography probe or upper gastrointestinal endoscopy, and prolonged tracheal intubation for more than two days should be the risk factors causing arytenoid cartilage dislocation. And calcification of the laryngeal cartilage and morphological changes of the cervical vertebrae along with aging might also contribute to dislocating the arytenoid cartilage."
			Non-AD, 9674	52		4876 (50.3)	Abdominal/general surgery = 1807; Otorhinolaryngology, head and neck surgery = 1309; Orthopedics surgery = 1670; Obstetrics and gynecology = 967; Maxillo-facial and reconstructive surgery = 760; Cardiovascular surgery = 655; Neurosurgery = 659; Urology and adrenal gland surgery = 559; Ophthalmology = 361; Breast surgery = 196; Respiratory surgery = 359; Others = 372				NR					NA	NA	
Shen et al., 2014 [36]	Case-control study	China	AD, 16	51.7 ± 19.8	22.53±3.65	8 (50%)	Abdominal/general surgery = 11 (68.75%); Cardiovascular surgery = 3 (18.75%); Orthopedics surgery = 1 (6.25%); Neurosurgery = 1 (6.25%)	7 mm = 8 (50%); 7.5 mm = 8 (50%)	NR	NR	Video-laryngoscope	NR	NR	ASA I-II = 12 (75%); ASA III- IV = 4 (25%)	11 (68.75%)	NR	Left = 10 (62.5%); Right = 6 (37.5%)	"Non-smoking and anemic patients may be susceptible to postoperative arytenoid dislocation. However, neither of them was an independent risk factor for postoperative arytenoid dislocation."
			Non-AD, 16	58.63 ± 9.01	24.38 ± 3.51	10 (62.5%)	Abdominal/general surgery = 11 (68.75%); Cardiovascular surgery = 3 (18.75%); Orthopedics surgery = 1 (6.25%); Neurosurgery = 1 (6.25%)	7 mm = 8 (47.5%); 7.5 mm = 10 (62.5%)			NR			ASA I-II = 16 (100%)	8 (50%)	NA	NA	
Tsuru et al., 2017 [17]	Retrospective cohort study	Japan	AD, 14	NR	NR	NR	Cardiovascular = 14 (100%)	NR	NR	NR	Conventional laryngoscope or Fiberoptic laryngoscopy	NR	NR	NR	NR	NR	NR	"The present study demonstrated that major cardiovascular operation is one of the significant risk factors leading to this complication."
			Non-AD, 19423	56 ± 16			Major surgery = 1374 (7%); Cardiothoracic surgery = 18059 (93%)				Conventional laryngoscope or Fiberoptic laryngoscopy					NA	NA	
Wu et al., 2018 [8]	Case-control study	China	AD, 26	54 ± 17.4	23.50 ± 3.69	15 (57.7%)	Abdominal/general surgery = 17 (65.5%); Cardiothoracic surgery = 4 (15.4%); Orthopedics surgery = 1 (3.8%); Gynecologic surgery = 1 (3.8%); Neurosurgery = 3 (11.5%)	7 mm = 11 (42.3%); 7.5 mm = 15 (57.7%)	4.03 ± 1.67	NR	Conventional laryngoscope or Fiberoptic laryngoscopy	NR	One attempt= 25 (96.2%); Three attempt = 1 (3.8%)	ASA I = 8 (30.8%); ASA II = 15 (57.7%); ASA III = 2 (7.7%); ASA IV = 1 (3.8%)	10 (38.46%)	NR	Left = 15 (57.7%); Right = 11 (42.3%)	"The use of an intubation stylet for endotracheal intubation appears to protect against AD. Prolonged operation time increases the risk of AD. These factors should be considered when assessing the risks of AD associated with endotracheal intubation and in efforts to avoid this complication."
			Non-AD, 78	54 ± 17.4	23.19 ± 3.35	45 (57.7%)	NR	NR	2.60 ± 1.51		Conventional laryngoscope or Fiberoptic laryngoscopy	NR	One attempt= 75 (96.15%); One attempt > 1 = 3 (3.85%)	NR	50 (64.1%)	NA	NA	
Yamanaka et al., 2009 [9]	Prospective cohort study	Japan	AD, 3	53	NR	NR	NR	NR	NR	NR	Conventional laryngoscope or Video-laryngoscope	NR	NR	ASA I or II = 3 (100%)	NR	NR	NR	"The age of the patient and duration of intubation were significant factors in the duration of hoarseness after tracheal intubation. In addition, the incidence of arytenoid cartilage dislocation was 0.097%."
			Non-AD, 3090	53		1356 (44%)		8 mm =1356 (44%); 7.5 mm = 1734 (56%)		283 ± 172	Conventional laryngoscope or Video-laryngoscope			ASA I or II		NA		
Yan et al., 2023 [18]	Case-control study	China	AD, 16	53.3 ± 10.8	24.64 ± 4.4	12 (75%)	Abdominal/general surgery = 14 (87.5%); Orthopedics surgery = 2 (12.5%)	NR	4.81 ± 1.9	NR	Conventional laryngoscope	Supine= 14 (87.5%); Prone = 2 (12.5%)	NR	NR	NR	NR	Left = 12 (75%); Right = 4 (25%)	"Postoperative AD incidence was significantly elevated in patients undergoing liver transplantation. This finding should be clinically relevant and alarming for anesthesiologists and clinicians to help avoid arytenoid dislocation and improve patient outcomes. Further studies that incorporate detailed data are needed to determine risk factors for AD."
			Non-AD, 30138	55 ± 15.7	24.82 ± 5.7	15222 (50.7%)	Liver Transplantation = 247 (0.8%)		3.33 ± 2.2		Conventional laryngoscope	Supine= 28406 (94.25%); Prone = 1732 (5.75%)				NA	NA	

Risk of Bias

The reviewers used study rating tools to evaluate the quality of each study, categorizing them as “good,” “fair,” or “poor” based on various criteria within the NIH tool. A “good” study is deemed to have the least risk of bias and produces valid results, while a “fair” study may have some bias but not to the extent of invalidating its findings; however, studies in this category exhibit varying strengths and weaknesses. Conversely, a “poor” rating indicates a significant risk of bias, typically leading to exclusion from the body of evidence unless no alternative evidence is available. Overall, there were six case-control studies of fair quality and one of good quality. Additionally, all cohort studies were relatively quality (Tables 2-3).

**Table 2:** NIH quality assessment tool for observational case-control studies NIH: National Institutes of Health

Study ID	1. Was the research question or objective in this paper clearly stated and appropriate?	2. Was the study population clearly specified and defined?	3. Did the authors include a sample size justification?	4. Were controls selected or recruited from the same or similar population that gave rise to the cases (including the same timeframe)?	5. Were the definitions, inclusion and exclusion criteria, algorithms, or processes used to identify or select cases and controls valid, reliable, and implemented consistently across all study participants?	6. Were the cases clearly defined and differentiated from controls?	7. If less than 100 percent of eligible cases and/or controls were selected for the study, were the cases and/or controls randomly selected from those eligible?	8. Was there use of concurrent controls?	9. Were the investigators able to confirm that the exposure/risk occurred prior to the development of the condition or event that defined a participant as a case?	10. Were the measures of exposure/risk clearly defined, valid, reliable, and implemented consistently (including the same time period) across all study participants?	11. Were the assessors of exposure/risk blinded to the case or control status of participants?	12. Were key potential confounding variables measured and adjusted statistically in the analyses? If matching was used, did the investigators account for matching during the study analysis?	Total scores: Yes = 1 // No = 0.5 // NR & NA & CD = 0	Grading
	Yes/No/Not reported (NR) or cannot determine (CD) or not applicable (NA)	Yes/No/Not reported (NR) or cannot determine (CD) or not applicable (NA)	Yes/No/Not reported (NR) or cannot determine (CD) or not applicable (NA)	Yes/No/Not reported (NR) or cannot determine (CD) or not applicable (NA)	Yes/No/Not reported (NR) or cannot determine (CD) or not applicable (NA)	Yes/No/Not reported (NR) or cannot determine (CD) or not applicable (NA)	Yes/No/Not reported (NR) or cannot determine (CD) or not applicable (NA)	Yes/No/Not reported (NR) or cannot determine (CD) or not applicable (NA)	Yes/No/Not reported (NR) or cannot determine (CD) or not applicable (NA)	Yes/No/Not reported (NR) or cannot determine (CD) or not applicable (NA)	Yes/No/Not reported (NR) or cannot determine (CD) or not applicable (NA)	Yes/No/Not reported (NR) or cannot determine (CD) or not applicable (NA)		
Hung et al., 2019 [20]	Yes	Yes	NR	Yes	Yes	Yes	Yes	Yes	Yes	Yes	NR	NR	9	Fair
Kong et al., 2022 [30]	Yes	Yes	Yes	Yes	Yes	Yes	NA	Yes	Yes	Yes	NR	NR	9	Fair
Lou and Lin, 2017 [34]	Yes	Yes	No	No	Yes	Yes	NA	Yes	Yes	Yes	NR	NR	8	Fair
Lou et al., 2023 [33]	Yes	Yes	Yes	Yes	Yes	Yes	NA	Yes	Yes	Yes	NR	NR	9	Fair
Shen et al., 2014 [36]	Yes	Yes	No	Yes	Yes	Yes	Yes	Yes	Yes	Yes	NR	NR	9.5	Good
Wu et al., 2018 [8]	Yes	Yes	NR	Yes	Yes	Yes	Yes	Yes	Yes	Yes	NR	NR	9	Fair
Yan et al., 2023 [18]	Yes	Yes	Yes	Yes	Yes	Yes	NA	Yes	Yes	Yes	NR	NR	9	Fair

**Table 3:** NIH quality assessment tool for observational cohort studies NIH: National Institutes of Health

ID	1. Was the research question or objective in this paper clearly stated?	2. Was the study population clearly specified and defined?	3. Was the participation rate of eligible persons at least 50%?	4. Were all the subjects selected or recruited from the same or similar populations (including the same time period)? Were inclusion and exclusion criteria for being in the study prespecified and applied uniformly to all participants?	5. Was a sample size justification, power description, or variance and effect estimates provided?	6. For the analyses in this paper, were the exposure(s) of interest measured prior to the outcome(s) being measured?	7. Was the time frame sufficient so that one could reasonably expect to see an association between exposure and outcome if it existed?	8. For exposures that can vary in amount or level, did the study examine different levels of the exposure as related to the outcome (e.g., categories of exposure, or exposure measured as continuous variable)?	9. Were the exposure measures (independent variables) clearly defined, valid, reliable, and implemented consistently across all study participants?	10. Was the exposure(s) assessed more than once over time?	11. Were the outcome measures (dependent variables) clearly defined, valid, reliable, and implemented consistently across all study participants?	12. Were the outcome assessors blinded to the exposure status of participants?	13. Was the loss to follow-up after baseline 20% or less?	14. Were key potential confounding variables measured and adjusted statistically for their impact on the relationship between exposure(s) and outcome(s)?	total scores	
	Yes/No/Not reported (NR) or cannot determine (CD) or not applicable (NA)	Yes/No/Not reported (NR) or cannot determine (CD) or not applicable (NA)	Yes/No/Not reported (NR) or cannot determine (CD) or not applicable (NA)	Yes/No/Not reported (NR) or cannot determine (CD) or not applicable (NA)	Yes/No/Not reported (NR) or cannot determine (CD) or not applicable (NA)	Yes/No/Not reported (NR) or cannot determine (CD) or not applicable (NA)	Yes/No/Not reported (NR) or cannot determine (CD) or not applicable (NA)	Yes/No/Not reported (NR) or cannot determine (CD) or not applicable (NA)	Yes/No/Not reported (NR) or cannot determine (CD) or not applicable (NA)	Yes/No/Not reported (NR) or cannot determine (CD) or not applicable (NA)	Yes/No/Not reported (NR) or cannot determine (CD) or not applicable (NA)	Yes/No/Not reported (NR) or cannot determine (CD) or not applicable (NA)	Yes/No/Not reported (NR) or cannot determine (CD) or not applicable (NA)	Yes/No/Not reported (NR) or cannot determine (CD) or not applicable (NA)		
Jang et al., 2021 [13]	Yes	Yes	Yes	Yes	No	Yes	NA	NA	NA	NA	Yes	No	Yes	NR	8	Fair
Jiang et al., 2023 [29]	Yes	Yes	Yes	Yes	No	Yes	NA	NA	NA	NA	Yes	No	Yes	NR	8	Fair
Lee et al., 2015 [31]	Yes	Yes	Yes	Yes	No	Yes	NA	NA	NA	NA	Yes	No	Yes	NR	8	Fair
Liu et al., 2018 [32]	Yes	Yes	Yes	Yes	No	Yes	NA	NA	NA	NA	Yes	No	Yes	NR	8	Fair
Lou et al., 2018 [15]	Yes	Yes	CD	Yes	Yes	Yes	No	NA	NA	NA	Yes	No	Yes	NR	8	Fair
Rubin et al., 2005 [25]	Yes	Yes	CD	Yes	Yes	Yes	No	NA	NA	NA	Yes	No	Yes	NR	8	Fair
Saigusa et al., 2013 [35]	Yes	Yes	Yes	Yes	No	Yes	NA	NA	NA	NA	Yes	No	Yes	NR	8	Fair
Tsuru et al., 2017 [17]	Yes	Yes	Yes	Yes	Yes	Yes	NA	NA	NA	NA	Yes	No	Yes	NR	8.5	Fair
Yamanaka et al., 2009 [9]	Yes	Yes	Yes	Yes	No	Yes	NA	NA	NA	NA	Yes	No	Yes	NR	8	Fair

Results of Syntheses

**Figure 2:**
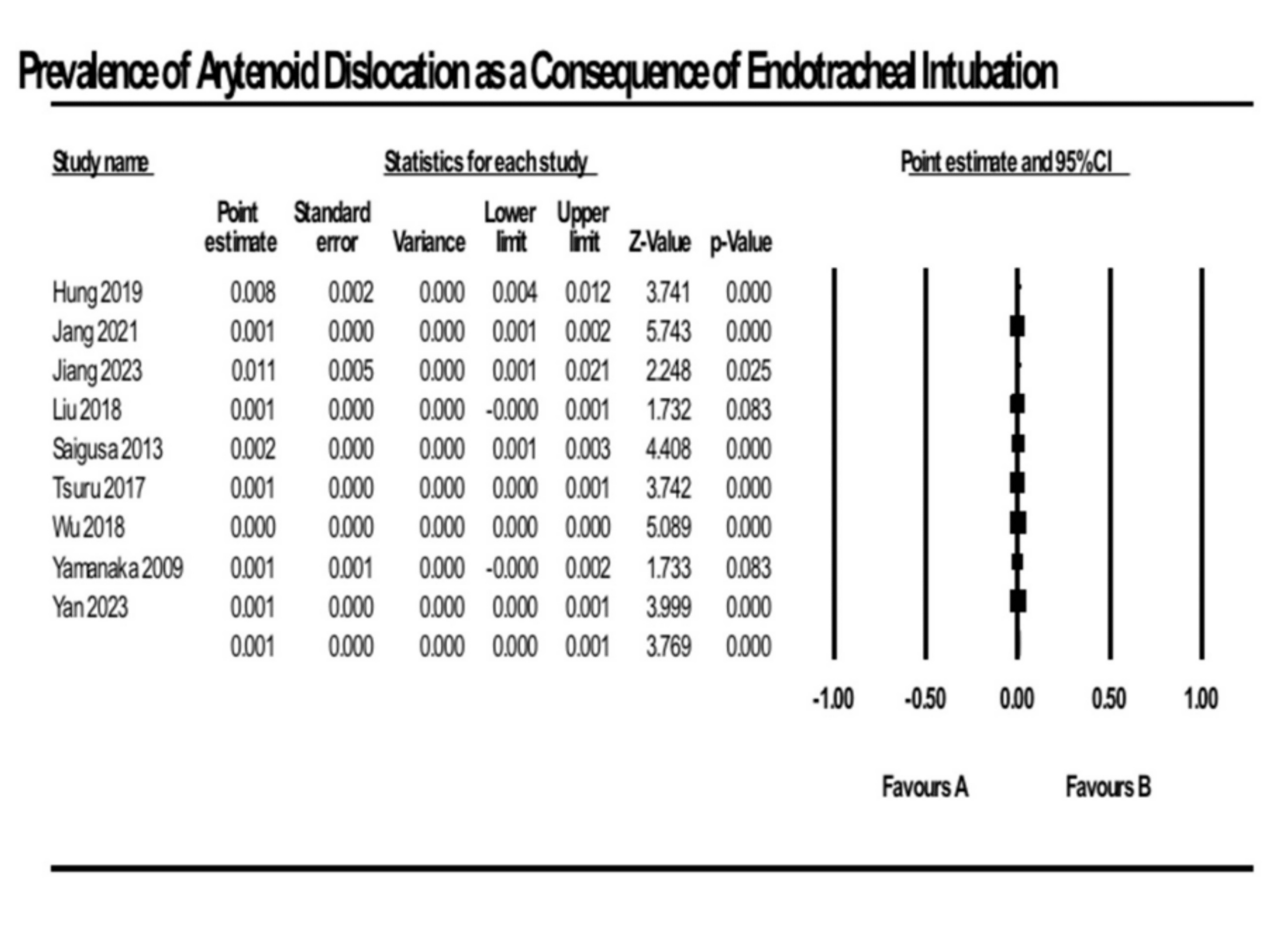
Flow chart of risk of bias in selected studies References [20,13,29,32,35,17,8,9,18]

The prevalence of AD was assessed using a random-effects model, incorporating data from nine studies. The estimated pooled prevalence was 0.093%, with a 95% CI ranging from 0.045% to 0.14%. Considerable heterogeneity was observed; the I² value was 91%, with a Q-value of 89.14 and a p-value < 0.001, suggesting substantial variability among the studies (Figure 2).

Risk Factors for AD

We identified multiple factors associated with AD across the included studies. Older age was linked to prolonged hoarseness post-intubation, whereas younger age and female gender increased AD risk. Additionally, taller stature and higher BMI were highlighted as predisposing factors for AD. Specific surgical procedures, such as malar reduction combined with mandible angle ostectomy, liver transplantation, primary cardiovascular operations, and abdominal surgery, were associated with an elevated risk of AD. Procedures involving esophageal instrumentation, such as transesophageal echocardiography and nasogastric tube insertion, also heightened AD risk. Prolonged surgical and anesthesia durations were significant contributors to AD risk, potentially due to extended intubation and airway manipulation. Head-neck movement during surgery, especially in head and neck procedures, increases the risk of AD due to altered patient positioning.

Moreover, residents in standardized training, particularly first-year anesthesia residents, demonstrated an increased risk of AD. Conversely, complex intubation cases were associated with a higher likelihood of AD. The use of an intubation style significantly reduced the risk of surgery-associated AD (see Table 1).

Discussions

AD is a rare complication following endotracheal intubation, with multiple risk factors and severe consequences if left untreated. In a pooled sample from nine studies, the incidence of AD was found to be very low, at 0.09%. This represents the first accurate incidence estimate in the literature, with a standard deviation of 0.02%. There was considerable variability in reported incidences, resulting in high heterogeneity. For example, Yamanaka et al. and Wu et al. reported incidences as low as 0.01%, while Jiang et al. reported an incidence of 1.1% [8,9,29]. This variability might stem from underdiagnosis in larger datasets, as AD may spontaneously recover with improved symptoms [14,20,38,39]. Additionally, some cases of AD may be mistaken for recurrent laryngeal nerve paralysis [12,14,20].

Several studies identified risk factors for AD, though there was considerable variation among different cohorts. Lee et al. found that AD occurred even with a clear glottic view during laryngoscopy, suggesting that factors beyond intubation difficulty may contribute [31]. Researchers investigated age and gender as potential risk factors for AD. Yamanaka et al. found that gender did not contribute to a longer duration of hoarseness after tracheal intubation, but older age did [9]. Jang et al. found that younger age and female gender were associated with an increased risk of AD [13]. Hung et al. reported that patients undergoing bariatric surgery, especially younger patients, may be at a higher risk of AD [20]. Conversely, Jiang et al. and Wu et al. noted that demographic characteristics such as age and gender did not show statistical significance when comparing data between non-dislocation and dislocation groups [8,29].

Anthropometric indices may raise the risk of AD. Lou et al. identified BMI as a significant independent risk factor for AD, whereas Yamanaka et al. and Wu et al. did not [8,9,15]. BMI may be associated with an increased risk of AD due to several factors [13,15]. The increased pressure exerted by the endotracheal tube in the arytenoid cartilage region, combined with a longer duration of anesthesia, can predispose individuals with a higher BMI to AD [30]. Moreover, a higher BMI has been linked to an increased risk of joint dislocations in various medical contexts [40,41]. Morbid obesity (BMI of 40 or more) is a specific risk factor for joint dislocations, which may extend to AD as well [41]. The additional soft tissue impingement following surgical procedures in obese patients can further contribute to the risk of dislocation [40]. Tsuru et al. also identified patient height as an unexpected risk factor, though the authors did not provide a clear explanation for this finding [17].

Specific surgical procedures have been linked to a higher risk of AD. In studies by Jiang et al. and Yan et al., surgeries like combined malar reduction with mandible angle ostectomy and liver transplantation were linked to a higher risk of AD [29,37]. Similarly, Tsuru et al. and Saigusa et al. found that major cardiovascular surgeries significantly contribute to arytenoid cartilage dislocation [17,35]. Additionally, Kong et al. revealed that abdominal surgery was a significant risk factor for AD in their study [30].

Several studies have demonstrated a link between the duration of both the operation and anesthesia and a higher incidence of AD. Wu et al. found a notable association between longer operation durations and increased risk of AD, with a factor increase of 1.89 for each additional hour of operation time [8]. Lee et al. similarly noted that prolonged surgical durations may elevate the risk of AD, potentially due to extended intubation and airway manipulation [31]. Kong et al. corroborated these findings, showing a significantly longer median operative time in patients with AD than those without, with an operative time exceeding three hours identified as a risk factor [30]. Hung et al. also observed longer anesthesia durations in patients undergoing bariatric surgery, supporting the notion that extended periods under anesthesia may contribute to the risk of AD [20].

Conversely, Yamanaka et al. and Saigusa et al. found that the duration of intubation is a significant risk factor for AD. Yamanaka et al. reported that longer intubation periods resulted in a longer duration of hoarseness after surgery [9]. Saigusa et al. found that the majority of patients with arytenoid cartilage dislocation underwent extubation more than two days post-surgery [35].

Additional factors that may elevate the risk of AD include head position during surgery, difficult intubation, and the experience level of residents performing the procedure. Head position during surgery emerged as a significant risk factor for AD, as indicated by findings from Jiang et al., Lee et al., and Jang et al. Patients who experienced head-neck movement during surgery, particularly those undergoing head and neck procedures with changes in patient positioning, such as rotation or extension of the head, were more predisposed to developing AD [13,29,31]. Despite not achieving statistical significance, residents in standardized training showed the potential to increase the risk of this complication [29]. Additionally, Jang et al. noted that first-year anesthesia residents were more frequently involved in intubations leading to AD [13]. Cases characterized by difficult intubation are more likely to develop AD [17]. Wu et al. demonstrated that intubation significantly reduced the risk of surgery-associated AD compared to cases without its use [8].

This systematic review has limitations, including the reliance on patient complaints and subjective judgments for diagnosing hoarseness in many studies, which may have led to the underdiagnosis of AD. Additionally, the inclusion of retrospective studies introduces the possibility of selection bias, which affects the generalizability of the findings. Furthermore, the scarcity of studies focusing on individual risk factors limited the suitability of the data for comprehensive analysis, highlighting the need for more targeted research in this area.

## Conclusions

AD is a rare but potentially severe complication following endotracheal intubation, with a CI of 0.09%. Variability in reported incidences may stem from underdiagnosis in larger datasets. Multiple risk factors for AD have been identified, including age, gender, anthropometric measures such as BMI, specific surgical procedures, duration of surgery and anesthesia, head position during surgery, difficult intubation, and the experience level of medical personnel performing intubations. However, limitations such as reliance on subjective diagnoses and retrospective study designs necessitate further research to understand better and mitigate the risk factors associated with AD.
